# Chemical profiling and biological activities of the medicinal macrofungus *Coriolopsis gallica*: volatile, non-volatile, and fatty acid composition with antioxidant and in silico–supported anti-inflammatory properties

**DOI:** 10.1007/s40199-026-00600-6

**Published:** 2026-03-28

**Authors:** Roukia Zatout, Ouided Benslama, Chaima Zatout, Stefania Garzoli

**Affiliations:** 1 Blida 1 University, Blida, Algeria; 2 Larbi Ben M’hidi University of Oum El Bouaghi, Oum el Bouaghi, Algeria; 3 Constantine 1 University, Constantine, Algeria; 4 Sapienza University of Rome, Rome, Italy

**Keywords:** Coriolopsis gallica, Antioxidant activity, Anti-inflammatory activity, Chemical composition, Fatty acids, Molecular docking, COX-2 and 5-LOX

## Abstract

**Background:**

*Coriolopsis gallica*, a medicinal macrofungus, was collected from a decaying hardwood trunk in Constantine, Algeria.

**Objective:**

*C. gallica* was investigated to explore its chemical composition and biological activities.

**Methods:**

Morphological analysis confirmed characteristic features of *C. gallica* and GC-MS and in silico analyses were performed.

**Results:**

Chemical profiling revealed that the volatile fraction was dominated by benzyl benzoate (16.6%), and 2,4-di-tert-butylphenol (14.4%), while the non-volatile fraction was primarily lactose (90.6%) with notable aucubin (4.3%), a bioactive iridoid glycoside. Fatty acid analysis indicated high content of linoleic acid (79.0%) and oleic acid (11.8%), suggesting potential anti-inflammatory and antioxidant effects. The ethanolic extract demonstrated significant antioxidant activity in the DPPH assay (IC₅₀ = 11.28 ± 0.24 µg/mL) and moderate anti-denaturation activity (IC₅₀ = 6.75 ± 0.5 mg/mL), consistent with the presence of bioactive metabolites. Molecular docking analyses of all identified metabolites against COX-2 and 5-LOX revealed a range of predicted binding affinities and interaction patterns, with several fatty acids, including linoleic and oleic acids, showing favorable docking scores, while aucubin and phenolic compounds exhibited complementary hydrogen-bonding and hydrophobic interactions. These in silico results provide comparative, hypothesis-generating insights that complement the in vitro antioxidant and anti-denaturation assays.

**Conclusion:**

Overall, the combined chemical, biological, and computational analyses highlight *Coriolopsis gallica* as a promising natural source of structurally diverse bioactive compounds with antioxidant and anti-inflammatory relevance, supporting its interest in early-stage natural product research.

## Introduction

 Macrofungi have long been recognized as valuable sources of bioactive compounds with promising nutritional and medicinal properties [1, 2]. Among them, macrofungi belonging to the family Polyporaceae are known for producing a wide array of secondary metabolites, including polysaccharides, terpenoids, phenolic compounds, and fatty acids, which contribute to their diverse pharmacological activities [3, 4]. These compounds exhibit antioxidant, antimicrobial, anti-inflammatory, and anticancer effects, supporting the traditional use of many polypores in folk medicine [5, 6].

The genus *Coriolopsis* (Polyporaceae, Basidiomycota) comprises lignicolous (wood-decaying) fungi commonly found on dead hardwoods in tropical, subtropical, and temperate regions [7]. These species play an important ecological role in lignin degradation and carbon cycling and represent a valuable reservoir of secondary metabolites with pharmacological potential. Several species, including *C. polyzona*, *C. caperata*, *C. rigida*, *C. gallica*, *C. strumosa*, and *C. telfairii*, have attracted attention for their diverse chemical composition and biological activities [8–10].

*Coriolopsis* species produce a wide range of volatile organic compounds (VOCs), such as alcohols, aldehydes, ketones, terpenes, and aromatic hydrocarbons, that contribute to their aroma and may exhibit antimicrobial or antioxidant effects. For instance, *C. polyzona* emits 1-octen-3-ol, benzaldehyde, and nonanal, while *C. rigida* and *C. strumosa* generate terpenoid-rich volatiles with notable antimicrobial properties [11, 12]. Their non-volatile constituents include phenolic compounds, triterpenoids, flavonoids, and polysaccharides, many of which display antioxidant, cytotoxic, and immunomodulatory activities. Extracts from *C. polyzona* and *C. caperata* have shown strong radical-scavenging potential, and polysaccharides from *C. gallica* demonstrate immunostimulatory and anticancer effects [13–15].

In addition, fatty acids represent another important group of bioactive molecules in *Coriolopsis* species, contributing to both their nutritional value and pharmacological potential [16–18]. Previous studies have shown that members of this genus typically contain a high proportion of unsaturated fatty acids, particularly linoleic (C18:2), oleic (C18:1), and palmitic (C16:0) acids [19, 20]. These fatty acids are known to modulate oxidative stress and inflammatory responses by influencing lipid peroxidation processes and inflammatory mediator production, thereby contributing to their antioxidant and anti-inflammatory effects [17, 21]. Moreover, linoleic acid, serves as a biochemical precursor of 1-octen-3-ol—a volatile compound responsible for the characteristic “mushroom aroma” and also implicated in antioxidant and cellluar signaling activities [22].

At the molecular level, increasing evidence indicates that fungal-derived phenolic compounds, terpenoids, and fatty acids may exert anti-inflammatory effects through the modulation of key enzymes involved in eicosanoid biosynthesis, particularly cyclooxygenase-2 (COX-2) and 5-lipoxygenase (5-LOX). Inhibition of these enzymes can reduce the production of pro-inflammatory mediators such as prostaglandins and leukotrienes, thereby attenuating inflammatory responses. In parallel, these compounds may contribute to inflammation control through their antioxidant capacity by limiting reactive oxygen species (ROS)-mediated signaling pathways [23].

Despite these findings, *Coriolopsis gallica* remains relatively underexplored, particularly in North Africa. Algeria, with its rich biodiversity and varied ecological conditions, harbors numerous wild macrofungi that have not been chemically or biologically characterized. Investigating these native species may reveal novel natural compounds with therapeutic potential.

This study aims to comprehensively characterize the Algerian medicinal macrofungus *Coriolopsis gallica* by analyzing its volatile, non-volatile, and fatty acid compositions, and by evaluating its antioxidant activity alongside in in silico-supported anti-inflammatory potential, targeting COX-2 and 5-LOX enzymes, thereby contributing to its development as a natural source of bioactive compounds for pharmaceutical and nutraceutical applications.

## Materials and methods

### Fungal material

The fungal material used in this study consisted of fruiting bodies of *C. gallica*, collected in February 2025 from decaying hardwood logs in the forested area of the Constantine region, Algeria. Samples were authenticated based on macroscopic characteristics using standard taxonomic keys, and the identification was verified by comparison with descriptions available in relevant mycological literature [24]. The fruiting bodies were air-dried in the shade, stored in airtight containers, ground into a fine powder, and preserved for further analyses.

A representative voucher specimen has been deposited at the Herbarium of the Department of Pharmacy, Université Constantine 3 – Salah Boubandire, under the voucher number MC04-2025-001, where it is permanently stored for future reference.

### Extraction of *Coriolopsis gallica*

The extraction of the macrofungus *C. gallica* was carried out using 96% ethanol as the solvent. Approximately 20 g of dried fungal material was macerated in 200 mL of 96% ethanol (1:10, w/v) at room temperature for 48 h with occasional stirring [25]. The extraction was repeated twice under the same conditions, and the resulting filtrates were combined and homogenized. The solvent was then evaporated under reduced pressure using a rotary evaporator at a temperature not exceeding 40 °C to obtain the crude ethanolic extract. The extract was transferred into airtight vials and stored at 4 °C until further phytochemical and biological analyses.

### Headspace solid phase microextraction (HS-SPME)

The volatile chemical composition of *C. gallica* dry powder was performed by HS-SPME sampling technique followed by GC-MS analysis [26]. About 0.5 g of the powder was placed inside a 4 mL glass vial with PTFE-coated silicone septum. The extraction process of volatiles was carried out on a SPME device from Supelco (Bellefonte, PA) with 1 cm fiber coated with 50/30µm DVB/CAR/PDMS (divinylbenzene/carboxen/polydimethylsiloxane). Before use, the fiber was conditioned at 270 °C for 30 min. The equilibration time was obtained heating to 60 °C for 15 min. After this time, the fiber was exposed to the headspace of the samples for 20 min at 60 °C to capture and concentrate the volatiles. Lastly, the analytes were desorbed thermally in the GC injector maintained at 250 °C for 2 min in splitless mode.

### GC-MS analysis

The analysis was carried out on Clarus 500 model Perkin Elmer (Waltham, MA, USA) gas chromatograph coupled with a mass spectrometer equipped. The capillary column was a Varian Factor Four VF-5. To characterize the volatile composition of the sample, the operative conditions were set as follows: from 45 °C to 220 °C at 6°/min and finally held for 15 min. Helium was used as carrier gas at a constant rate of 1 mL/min. MS scans were recorded within the range 35–450 m/z using EI ionization (energy 70 eV). Identification of compounds was based on the comparison of the mass spectra of pure components stored in the Wiley 2.2 and Nist 11 libraries database and on the comparison of the Linear Retention Indices (LRIs) calculated using a series of alkane standards (C_8_–C_25_ *n*-alkanes) with that available retention data reported in the literature. The relative amounts of the constituents were expressed as percentages (mean of three replicates) without the use of an internal standard and any factor correction.

### GC-MS analysis of *Coriolopsis gallica* after silanization

To describe the non-volatile fraction of *C. gallica*, a derivatization reaction was carried out. The analysis was performed using the same apparatus GC-FID/GC-MS and the same capillary column (Varian Factor Four VF-5). The applied program temperature was as follows: 70 °C then a gradient of 7 °C/minute up to 170 °C held for 1.0 min and a gradient of 8 °C/minute up to 250 °C held constant for 25 min. Mass spectra were acquired in electron impact mode. The identification of the compounds was performed considering the percentage of similarity with the mass spectra (MS) present in the instrument library database (NIST 11). Quantification was performed as described above (2.4.)

### GC-MS determination of fatty acids (FAs) content

Fatty acid content was determined by GC/MS technique after lipid extraction process and synthesis of FAs methyl esters. In detail, the samples (0.5 g) were dissolved with 10 mL of chloroform/methanol (2:1 v/v). Then, 1 mL of each extract was dried with nitrogen and transmethylated with BF_3_-CH_3_OH at 70 °C for 30 min. The extraction of the obtained fatty acid methyl esters (FAMEs) was carried out with n-hexane. 2 µL of the extract was injected into the column in splitless mode. The gas chromatographic conditions were as follows: the injector was set at 280 °C and the oven temperature program started from 170 °C and increased up to 260 °C with a rate of 3 °C/min and held constant for 15 min. Component identification and quantification were performed as previously described. The analyses were performed in triplicate.

### Antioxidant activity

The antioxidant capacity of *C. gallica* extracts was evaluated in vitro using the DPPH (2,2-diphenyl-1-picrylhydrazyl) free radical scavenging assay. Ascorbic acid (vitamin C) served as a standard reference for comparison. A 0.1 mM DPPH solution was freshly prepared by dissolving 0.91 mg of DPPH in 23 mL of absolute ethanol to ensure complete solubilization and maintain radical stability.

The fungal extract was initially dissolved in a 1:1 (v/v) mixture of 0.1 M acetate buffer (pH 5.5) and ethanol to improve solubility and promote uniform distribution. Starting from a stock solution of 8 mg/mL, serial two-fold dilutions were prepared to obtain a range of concentrations down to approximately 1/256th of the original stock.

For the assay, 100 µL of the DPPH solution was combined with 100 µL of each extract dilution in a 96-well microplate. Appropriate blanks and controls were included, and all samples were tested in triplicate. The plates were incubated at room temperature (25 ± 2 °C) in the dark for 30 min to prevent light-induced degradation of the radical.

After incubation, absorbance was measured at 517 nm using a UV–Vis microplate reader. Radical scavenging activity (RSA) was expressed as percentage inhibition, calculated using the formula:

$$\%\;inhibition\;=\;\left[\left(A_o-A_s\right)/A_o\right]\times100$$

where A₀ is the absorbance of the DPPH control and Aₛ is the absorbance in the presence of extract. Half-maximal inhibitory concentrations (IC₅₀) for the macrofungus *C. gallica* were determined graphically by plotting percentage inhibition against the logarithm of concentration and identifying the concentration corresponding to 50% inhibition. Vitamin C was assayed in parallel under identical conditions to benchmark the antioxidant performance of the tested plant extracts [27].

### Anti-denaturation activity

The anti-denaturation potential of the *C. gallica* ethanolic extract was evaluated using the bovine serum albumin (BSA) heat-denaturation method, following the procedure of Bailey-Shaw et al. (2017) [28] with slight modifications. A freshly prepared 0.4% (w/v) BSA solution in Tris-buffered saline (TBS) was adjusted to pH 6.4 using glacial acetic acid as necessary. The extract was dissolved in distilled water and tested at concentrations of 40, 20, 10, 5, 2.5, and 1.25 mg/mL. For each assay, 1.0 mL of BSA solution was mixed with an appropriate volume of extract solution to achieve the desired concentration, and the volume was adjusted to 2.5 mL with distilled water (total volume not exceeding 3.0 mL).

The negative control consisted of BSA with distilled water only, while aspirin at corresponding concentrations was used as the positive (reference) control. The reaction mixtures were incubated at 37 °C for 15 min and subsequently heated at 70 °C for 10 min in a water bath to induce denaturation. After cooling to room temperature for 20 min, the turbidity of each sample, reflecting protein aggregation, was measured at 660 nm using a UV–visible spectrophotometer.

The percentage inhibition of protein denaturation was calculated using the formula:

$$\%\;inhibition\;=\;\left[\left(A_{control}-A_{sample}\right)/A_{control}\right]\times100$$

where A _control_ is the absorbance of the negative control and A_sample_ is that of the extract-treated sample. All tests were conducted in triplicate, and results are expressed as mean ± SD to facilitate comparison of the anti-denaturation activity of *C. gallica* across different concentrations.

### Molecular docking

#### Molecular docking studies

Molecular docking simulations were performed to investigate the interactions between selected bioactive compounds from *Coriolopsis gallica* and two key inflammatory enzymes, COX-2 (PDB ID: 1PXX) and 5-LOX (PDB ID: 3V99). The docking workflow was carried out using the Molecular Operating Environment (MOE 2023.01) software [29].

#### Protein and ligand preparation

The crystal structures of COX-2 and 5-LOX were retrieved from the Protein Data Bank (PDB IDs: 1PXX and 3V99, respectively). Proteins were prepared by removing water molecules beyond 5 Å from the active site and adding hydrogen atoms. Partial charges and protonation states were assigned using MOE’s Protonate3D tool. The co-crystallized ligands, diclofenac (for COX-2) and arachidonic acid (for 5-LOX), were extracted from the crystal structures and prepared for docking. Additionally, the three selected bioactive compounds, 2,4-di-tert- butylphenol, aucubin, and linoleic acid, were obtained from PubChem and energy-minimized using the MMFF94x force field in MOE.

#### Redocking validation

To validate the docking protocol, redocking of the co-crystallized ligands into their respective active sites was performed. The root-mean-square deviation (RMSD) between the docked pose and the crystallographic conformation was calculated, yielding 0.814 Å for COX-2 and 1.235 Å for 5-LOX, confirming the reliability of the docking parameters.

#### Docking procedure

Docking simulations were conducted using MOE’s Dock module. The active site was defined based on the location of the co-crystallized ligand, and the Triangle Matcher placement method was applied, followed by London dG scoring for initial ranking. The top 100 poses for each ligand were refined using the GBVI/WSA dG scoring function. The final binding poses were analyzed for binding energies, hydrogen bonds, and hydrophobic interactions [30].

#### Visualization and analysis

The 3D and 2D interactions of the docked compounds with the enzymes were visualized using Discovery Studio and Pymol. Key interacting residues, hydrogen bond distances, and hydrophobic contacts were recorded for comparative analysis with the reference ligands.

### Statistical analysis

Analysis of variance (one-way and two-way ANOVA), followed by Tukey HSD post-hoc test test for multiple comparisons were carried out on the experimental data by means of XLSTAT software (Addinsoft SARL, New York, 412 NY, USA). Results were considered statistically significant when *p* ≤ 0.05.

## Results and discussion

### Morphological identification of *Coriolopsis gallica*

The specimen was morphologically identified as *Coriolopsis gallica* based on its perennial, sessile basidiocarps; finely tomentose brown upper surface; corky context; and a characteristic daedaloid to labyrinthine pore surface with 2–5 pores/mm.

The basidiomata were collected from a decaying hardwood trunk in Constantine (Algeria) in February 2025. The fungus developed as perennial, sessile, bracket-shaped basidiocarps arranged laterally on the host tree. The basidiocarps were applanate to slightly convex, with individual brackets measuring approximately 6–12 cm in width and 1–2.5 cm in thickness. The upper surface was dry, tough, and brown to yellow-brown, exhibiting a finely tomentose texture and weakly concentric zoning, with a rounded margin that appeared slightly lighter than the disc.

**Fig. 1:**
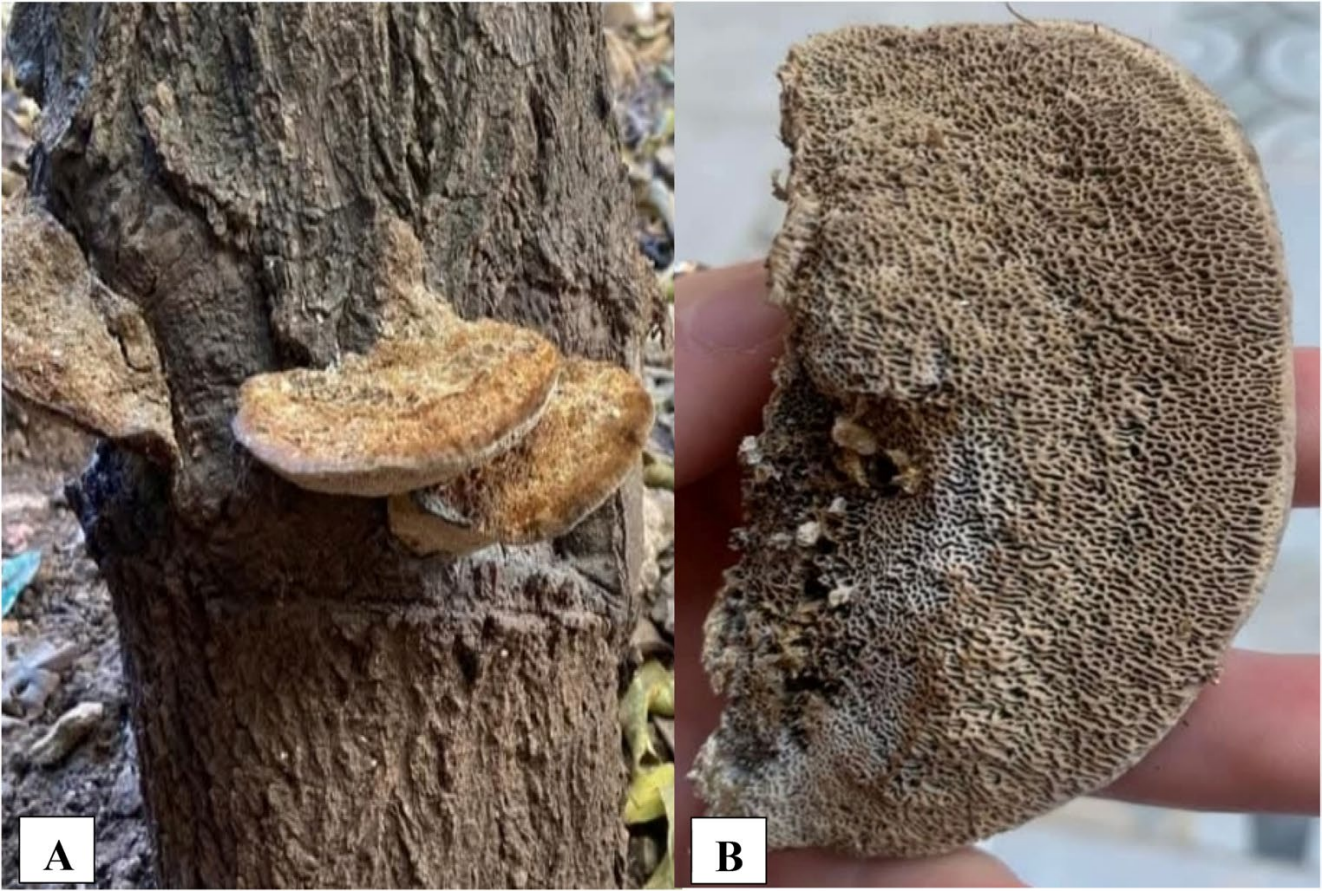
*Coriolopsis gallica* collected from a decaying hardwood trunk in Constantine, Algeria (February 2025), Photographed by M. Bouhedja. (**A**) Basidiocarps growing laterally on the host tree, showing perennial, sessile, bracket-shaped morphology. (**B**) Detail of the hymenial surface displaying characteristic irregular, daedaloid to labyrinthine pores

The hymenial surface displayed a characteristic poroid structure, composed of densely packed, irregular, maze-like pores (daedaloid to labyrinthine). The pores were small (typically 2–5 pores/mm) and elongated, corresponding closely to the diagnostic poroid configuration of the genus *Coriolopsis*. The pore layer was light beige to pale brown, consistent with mature, perennial basidiocarps. The context was corky and tough, with a homogeneous brown color typical of *Coriolopsis* species [31, 32]. The specimens were found growing directly on the trunk of a broad-leaf tree, a substrate preference characteristic of *Coriolopsis gallica*. This species is a well-known white-rot Basidiomycete capable of degrading lignin and cellulose in dead or weakened hardwood hosts [9, 33] (Fig. 1).

### Volatile chemical composition of *Coriolopsis gallica*

The volatile fraction of *C. gallica* was dominated by benzyl benzoate (48.1%) and 2,4-di-tert-butylphenol (41.7%) (Table 1). Minor components included linalool formate (5.7%), camphor (3.0%), and α-farnesene (1.6%). Among these, 2,4-di-tert-butylphenol stands out as a major compound with well-documented antioxidant, antimicrobial, and anti-inflammatory activities. Its relatively high abundance (41.7%) in *C. gallica* is particularly interesting, as this phenolic compound is rarely reported in basidiomycete fungi [34–36]. However, this compound could derive from accidental contamination and therefore it is not possible to demonstrate with absolute certainty that the 2,4-di-tert-butylphenol derives from the analyzed sample and therefore derives from a natural source. Benzyl benzoate, while primarily known for its insecticidal and soothing properties, may contribute mild antioxidant effects [37, 38]. Minor monoterpenoids such as camphor and linalool formate, have been reported to exert supportive antioxidant and anti-inflammatory (anti-denaturation) activities [39, 40]. Although α-farnesene’s direct biological effects are limited, it may act synergistically with other compounds. Collectively, these constituents likely contribute to the overall antioxidant and anti-inflammatory potential of the volatile extract.

**Table 1:** Volatile Chemical profile of *Coriolopsis gallica* (percentage mean values±standard deviation)

Number	Component	LRI^calc^	LRI^lit^	%
1	camphor	1142	1139	3.0 ± 0.09
2	linalool, formate	1210	1206	5.7 ± 0.15
3	α-farnesene	1502	1506	1.6 ± 0.08
4	2,4-di-tert-butylphenol	1510	1512	41.7 ± 4.15
5	benzylbenzoate	1740	1737	48.1 ± 3.85
	**Total**			100.0

The components are reported according to their elution order on apolar column; Linear Retention Indices measured on apolar column; Linear Retention indices from literature

### Non-volatile chemical composition of *Coriolopsis gallica*

The non-volatile fraction was mainly composed of lactose (90.6%), accompanied by aucubin (4.3%), D-mannitol (2.2%), D-turanose (1.9%), and D-lyxose (1.0%). The exceptionally high proportion of lactose suggests its role as a major carbohydrate reserve in *C. gallica*, potentially supporting energy metabolism and growth. Its abundance may also reflect specific enzymatic pathways involved in disaccharide accumulation in wood-decaying fungi [41, 42].

The identification of aucubin, an iridoid glycoside typically found in higher plants, represents a novel and noteworthy discovery in this fungal species [43]. Aucubin is known to exert anti-inflammatory, hepatoprotective, and antioxidant effects, which could significantly contribute to the pharmacological potential of *C. gallica* [44, 45]. The presence of mannitol and other natural sugars further supports the antioxidant and osmoprotective roles commonly observed in macrofungi [46, 47] (Table 2).

**Table 2:** Non-volatile chemical profile of *Coriolopsis gallica* (percentage mean values±standard deviation)

Number	Component (tentative identification)	%
1	D-mannitol	2.2 ± 0.58
2	D-lyxose	1.0 ± 0.06
3	aucubin	4.3 ± 0.75
4	lactose	90.6 ± 10.01
5	D-turanose	1.9 ± 0.11
	**Total**	100.0

### Fatty acid composition of *Coriolopsis gallica*

The fatty acid analysis indicated a predominance of linoleic acid (79.0%), followed by oleic acid (11.8%), palmitic acid (9.0%), and a trace amount of stearic acid (0.2%).

The high level of linoleic acid, a polyunsaturated fatty acid with well-established anti-inflammatory, antioxidant, and membrane-stabilizing properties, is remarkable and highlights its major contribution to the biological profile of *C. gallica* [48, 49]. Oleic acid, a monounsaturated fatty acid, is also recognized for its antioxidant and anti-inflammatory capacities, while palmitic and stearic acids serve structural and metabolic roles [50, 51].

The predominance of unsaturated fatty acids, especially linoleic and oleic acids, suggests that *C. gallica* possesses a lipid profile typical of species with potent biological and therapeutic potential, reinforcing its value as a natural source of health-promoting compounds.

The combination of volatile phenolic compounds, plant-like glycosides, and unsaturated fatty acids gives *C. gallica* a distinctive chemical fingerprint. The joint presence of 2,4-di-tert-butylphenol, aucubin, and linoleic acid likely underlies the moderate but significant anti-inflammatory and anti-denaturation activities observed experimentally [52]. These findings position *C. gallica* as a promising natural source of multifunctional bioactive molecules with potential applications in biomedicine and natural product research (Table 3).

**Table 3:** Fatty acids content of *Coriolopsis gallica* (percentage mean values±standard deviation)

Number	Fatty acids	LRI^calc^	LRI^lit^	%
1	palmitic acid, C16:0	1975	1973	9.0 ± 1.70
2	linoleic acid, C18:2*n*6	2155	2152	79.0 ± 6.85
3	oleic acid, C18:1*n*9	2172	2171	11.8 ± 1.52
4	staeric acid, C18:0	2176	2178	0.2 ± 0.02
	**Total**			100.0
	Saturated Fatty acids			9.2
	Unsaturated Fatty acids			90.8

The components are reported according to their elution order on apolar column (VF-5ms); Linear Retention Indices measured on apolar column; Linear Retention indices from literature

### Antioxidant ability

The antioxidant potential of *C. gallica* ethanolic extract was evaluated using the DPPH radical scavenging assay and compared with ascorbic acid as a reference (Table 4). The extract showed a notable radical scavenging capacity with an IC₅₀ value of 11.28 ± 0.24 µg/mL, whereas ascorbic acid exhibited stronger activity (3.19 ± 0.64 µg/mL). Statistical analysis revealed a significant difference between both samples (*p* ≤ 0.05). Despite being less potent than the standard, the extract demonstrated considerable antioxidant activity, likely due to its complex mixture of bioactive metabolites.

**Table 4:** Antioxidant activity (IC₅₀ µg/mL) of *Coriolopsis gallica* extract compared with ascorbic acid

Sample	IC_50_ (µg/mL)
Extract	11.28 ± 0.24^a^
Ascorbic acid	3.19 ± 0.64^b^

Values are expressed as mean ± SD (*n* = 3). Different letters indicate statistically significant differences (Tukey’s HSD test, *p* ≤ 0.05). Same letters mean no significant difference

The observed activity can be attributed to both volatile and non-volatile compounds. Phenolic derivatives and major lipidic constituents may contribute to radical scavenging and lipophilic antioxidant protection, particularly in preventing lipid peroxidation. Non-volatile metabolites such as iridoid glycosides (e.g., aucubin) and sugar alcohols (e.g., D-mannitol) are also known for their free radical scavenging and redox-balancing effects [53–56].

Furthermore, fatty acids, mainly linoleic and oleic acids, likely recognized for its capacity to quench reactive oxygen species and reduce oxidative stress, while saturated fatty acids contribute to maintaining membrane integrity [57].

Collectively, these results indicate that *C. gallica* is a rich source of structurally diverse antioxidant molecules, encompassing both hydrophilic and lipophilic components, supporting its potential as a natural antioxidant agent.

### Anti-inflammatory activity

The anti-denaturation potential of the *C. gallica* ethanolic extract was evaluated using the heat-induced bovine serum albumin (BSA) denaturation assay, a preliminary screening method for protein-level anti-inflammatory activity. The results are summarized in Table 5. The extract showed a concentration-dependent inhibition of protein denaturation, ranging from 15.2 ± 0.8 at 1.25 mg/mL to 65.4 ± 2.0 at 40 mg/mL, with an IC_50_ value of 6.75 ± 0.5 mg/mL. 6.75 ± 0.5. In comparison, the reference drug Aspirin consistently showed stronger inhibition at all tested concentrations, reaching 97.12 ± 2.3 at 40 mg/mL with an IC_50_ of 2.61 ± 0.2 mg/mL. Statistical analysis using Tukey’s HSD test revealed significant differences (*p* ≤ 0.05) both among the different extract concentrations and between treatments, confirming the superior efficacy of Aspirin as a standard NSAID.

These results suggest that the *C. gallica* extract possesses moderate anti-denaturation activity, indicating its potential anti-inflammatory effects. This activity is likely associated with the presence of bioactive metabolites such as linoleic acid, aucubin, and 2,4-di-tert-butylphenol, which are known to stabilize proteins and exert anti-inflammatory and antioxidant activities [55, 58]. Protein stabilization by these compounds may help prevent functional conformation and prevent aggregation, consistent with previous reports on other biologically active mushroom extracts [59, 60].

While BSA denaturation provides initial evidence for anti-inflammatory potential, it is important to note that this assay does not fully reflect enzymatic or cellular inflammatory pathways. Therefore, these findings should be interpreted as preliminary, highlighting the need for further mechanistic studies using cellular or in vivo models to confirm therapeutic relevance.

Overall, this study represents the first report of anti-denaturation activity for the ethanolic extract of *C. gallica*, emphasizing its potential as a natural modulator of inflammatory pathways. Although the extract does not reach the potency of conventional NSAIDs, its diverse bioactive composition suggests possible synergistic effects, warranting further investigation in cellular and in vivo models to confirm its therapeutic relevance.

**Table 5:** Percentage inhibition and IC_50_ values of *Coriolopsis gallica* ethanolic extract at different concentrations

% of inhibition							
Samples	1.25 mg/mL	2.5 mg/mL	5 mg/mL	10 mg/mL	20 mg/mL	40 mg/mL	IC_50_ (mg/mL)
Extract	15.2 ± 0.8^h^	22.3 ± 0.6^g^	40.5 ± 1.2^f^	47.6 ± 1.5^e^	55.8 ± 1.8^d^	65.4 ± 2.0^c^	6.75 ± 0.5^A^
Aspirin	33.4 ± 1.8^f^	48.96 ± 1.2^d^	64.65 ± 0.8^c^	70.89 ± 2.3^b^	77.8 ± 1. ^a^	97.12 ± 2.3^a^	2.61 ± 0.2^B^

Values are expressed as mean ± SD (*n* = 3). Different lowercase letters within the same row indicate significant differences among concentrations, while differnt uppercaseletters indicate significant differences between samples. Same letters indicate no significant difference (Tukey’s HSD test, *p* ≤ 0.05)

### Molecular docking

#### Molecular docking analysis

To further explore the possible molecular basis underlying the anti-inflammatory potential of *Coriolopsis gallica*, molecular docking simulations were performed against two key enzymes involved in the inflammatory cascade, namely cyclooxygenase-2 (COX-2) and 5-lipoxygenase (5-LOX), which are responsible for the biosynthesis of prostaglandins and leukotrienes, respectively.

All identified metabolites of *C. gallica*, including volatile, non-volatile, and fatty acid constituents, were initially subjected to molecular docking analyses against both targets. The resulting docking scores spanned a broad range of binding affinities, reflecting the chemical diversity of the extract. For clarity and comparative purposes, only the top-ranked compounds for each enzyme, based on predicted binding energies, were selected and discussed in detail, together with the corresponding co-crystallized reference inhibitors (diclofenac for COX-2 and arachidonic acid for 5-LOX).

**Fig. 2:**
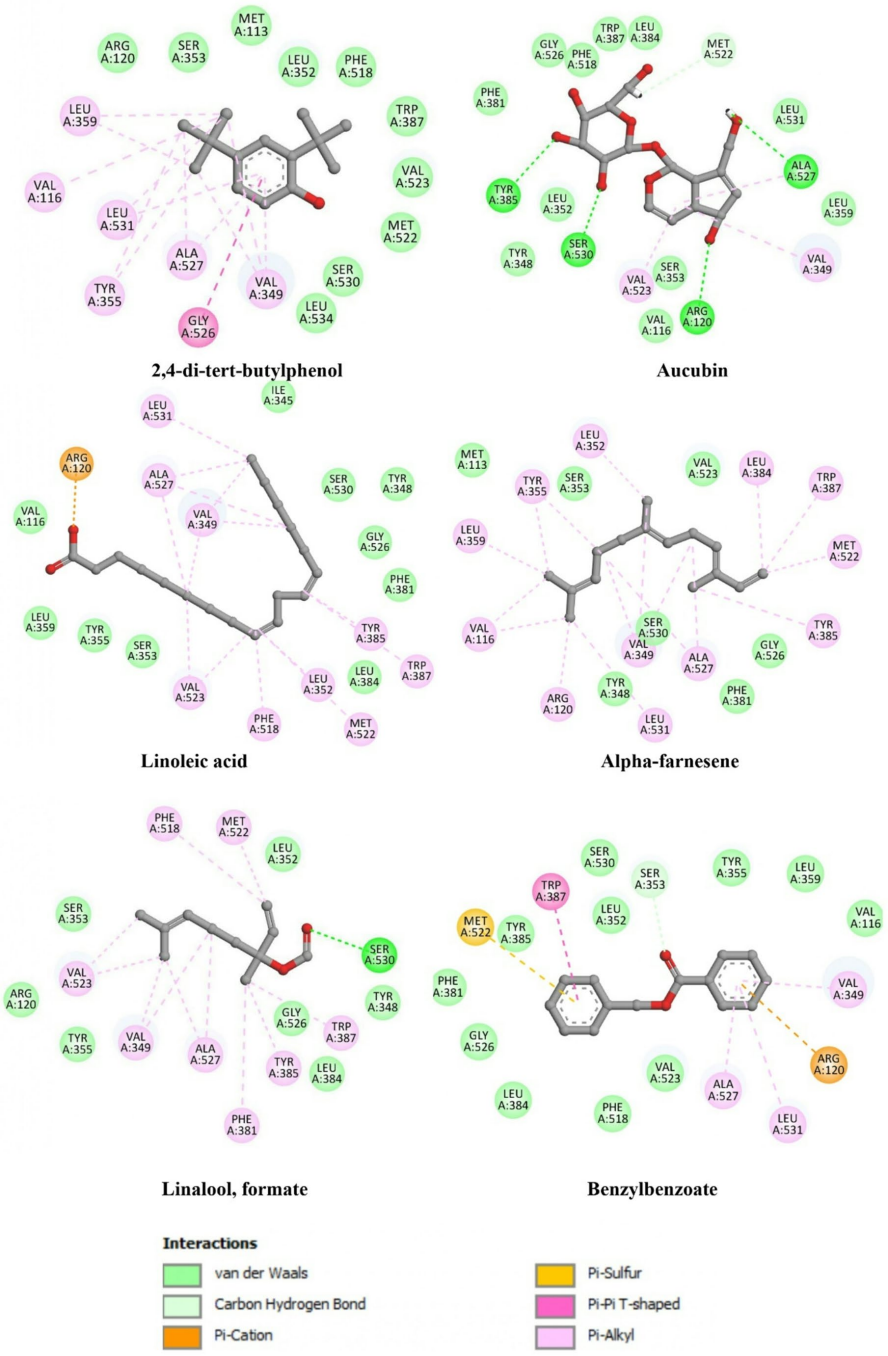
Two-dimensional interaction diagrams of the top-ranked *Coriolopsis gallica* metabolites docked into the active site of cyclooxygenase-2 (COX-2, PDB ID: 1PXX). The figure illustrates the predicted binding modes of 2,4-di-tert-butylphenol, aucubin, linoleic acid, α-farnesene, linalool formate, and benzyl benzoate within the COX-2 catalytic pocket

**Fig. 3:**
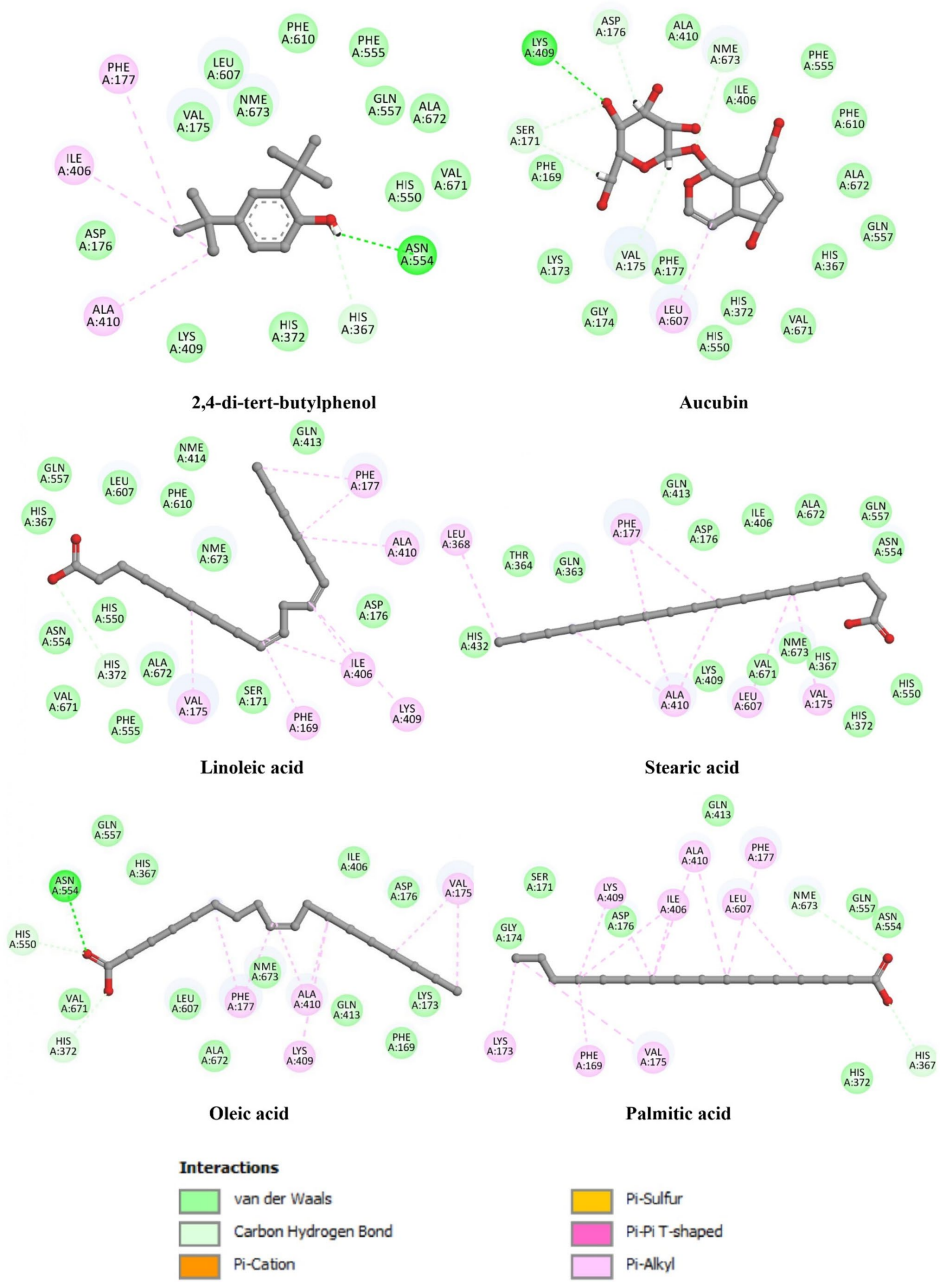
Two-dimensional interaction diagrams of the top-ranked *Coriolopsis gallica* metabolites docked into the active site of 5-lipoxygenase (5-LOX, PDB ID: 3V99). The figure depicts the predicted binding modes of 2,4-di-tert-butylphenol, aucubin, linoleic acid, oleic acid, stearic acid, and palmitic acid within the 5-LOX catalytic cavity

The docking results, summarized in Table 6 and illustrated in Figs. 2 and 3, highlight distinct and ligand-dependent interaction patterns within the active sites of COX-2 and 5-LOX, providing hypothesis-generating insights into potential ligand–enzyme interactions that may contribute to the observed in vitro anti-inflammatory effects.

**Table 6:** Binding energies, hydrogen bond interactions, and hydrophobic contacts of the selected *Coriolopsis gallica* compounds docked against COX-2 (1PXX) and 5-LOX (3V99)

			Binding energy (Kcal/mol)	Hydrogen interactions (Distance Å)	Hydrophobic interactions
COX-2 (1PXX)	Co-crystallized ligand	Diclofenac	−7.3	Tyr385 (1.87), Ser530 (2.13)	Val349, Val527
	Best docked compounds	2,4-di-tert-butylphenol	−6.1	-	Leu352, Trp387, Phe518, Gly526, Val349, Ala527, Tyr355
		Aucubin	−6.5	Tyr385 (2.75), Ser530 (2.34), Ser353 (2.60), Gly526 (2.22), Ala527 (2.32)	Ala527, Val349, Leu531
		Linoleic acid	−7.1	-	Ile377, Phe209, Leu534, Phe381, Phe205, Val349, Tyr348, Tyr345, Ala527, Val523, Leu352, Trp387, Arg120
		α-farnesene	−6.9	-	Leu356, Val116, Arg120, Leu531, Val349, Ala527, Tyr385, Met522, Trp387, Leu384, Leu352, Tyr352, Tyr355
		Linalool, formate	−6.3	Ser530 (2.64)	Met522, Phe518, Val523, Val349, Ala527, Phe381, Tyr385, Trp387
		Benzylbenzoate	−6.3	Ser353 (2.60)	Trp387, Val349, Leu531, Ala527, Arg120, Met522
5-LOX (3V99)	Co-crystallized ligand	Arachidonic Acid	−9.1	Arg596 (2.51), His600 (2.36)	Leu607, Phe177, Ala603, His600
	Best docked compounds	2,4-di-tert-butylphenol	−5.8	Asn554 (2.69), His367(2.60)	Phe177, Ile406, Lys409, Ala410
		Aucubin	−7.5	Lys409 (2.54), Ser171 (2.36, 2.48), Asp176 (2.62, 2.70), NME (2.24), Val175 (2.33)	Leu607
		Linoleic acid	−8.6	His372 (3.05)	Phe555, Phe610, Phe169, Val175, Lys173, Lys406
		Stearic acid	−8.4	-	Leu368, Phe177, Ala410, Leu607, Val175
		Oleic acid	−9.0	His550 (2.71), Asn554 (2.42), His372 (2.61)	Phe177, Ala410, Lys409, Val175
		Palmitic acid	−8.8	Nme673 (3.12), His367 (2.58)	Phe177, Leu607, Ala410, Ile406, Lys409, Val175, Phe169, Lys173

Inflammation is a complex biological response mediated by multiple enzymatic pathways, among which cyclooxygenase-2 (COX-2) and 5-lipoxygenase (5-LOX) play central and complementary roles [61, 62]. COX-2 is an inducible isoform of the cyclooxygenase family that catalyzes the conversion of arachidonic acid into prostaglandins, potent lipid mediators responsible for pain, swelling, and fever during the inflammatory response [63]. The overexpression of COX-2 is closely associated with chronic inflammatory disorders and is a major pharmacological target for nonsteroidal anti-inflammatory drugs (NSAIDs) such as diclofenac and celecoxib [64]. In parallel, 5-LOX catalyzes the oxidation of arachidonic acid to leukotrienes, another class of pro-inflammatory lipid mediators that promote leukocyte recruitment, vascular permeability, and tissue injury [65]. Dual inhibition of both COX-2 and 5-LOX pathways is therefore considered an effective strategy for achieving a broader and safer anti-inflammatory response, minimizing the side effects commonly associated with selective COX-2 inhibition [66]. The selection of these two enzymes as molecular targets in the present study thus provides a relevant mechanistic framework to understand how the bioactive compounds of *Coriolopsis gallica* may exert their anti-inflammatory effects at the molecular level. The molecular docking results presented in Table 6 demonstrate that the selected compounds from *C. gallica* exhibit variable binding affinities and interaction patterns toward the two key inflammatory targets, COX-2 and 5-LOX.

For COX-2 (PDB ID: 1PXX) (Fig. 2), the co-crystallized reference ligand diclofenac exhibited a binding energy of − 7.3 kcal/mol and formed stable hydrogen bonds with Tyr385 and Ser530, residues known to play a central role in ligand anchoring within the catalytic site.

Among the docked metabolites of *Coriolopsis gallica*, linoleic acid showed the most favorable predicted binding affinity (–7.1 kcal/mol), closely approaching that of diclofenac. Its binding mode was mainly stabilized through extensive hydrophobic interactions with residues involved in the accommodation of long-chain fatty acids, including Ile377, Phe381, Leu534, Val349, Tyr348, and Trp387. These interactions suggest a favorable spatial complementarity within the COX-2 active pocket.

α-Farnesene also displayed a comparable binding energy (–6.9 kcal/mol) and established multiple hydrophobic contacts with residues such as Leu356, Val116, Arg120, Leu531, Val349, Ala527, and Tyr385, reflecting the ability of hydrophobic terpenoid structures to occupy the enzyme cavity.

The iridoid glycoside aucubin exhibited a moderate binding affinity (–6.5 kcal/mol) but formed several hydrogen bonds with catalytically relevant residues, including Tyr385, Ser530, Ser353, Gly526, and Ala527, indicating a polar interaction profile that may contribute to ligand stabilization within the active site.

Similarly, 2,4-di-tert-butylphenol and benzyl benzoate showed moderate affinities (–6.1 and − 6.3 kcal/mol, respectively), predominantly stabilized by hydrophobic interactions with residues such as Leu352, Trp387, Phe518, Val349, and Ala527. Linalool formate, although exhibiting a slightly lower binding energy (–6.3 kcal/mol), engaged Ser530 through hydrogen bonding, suggesting an alternative interaction mode involving both polar and nonpolar contacts.

For 5-lipoxygenase (5-LOX, PDB ID: 3V99) (Fig. 3), the co-crystallized reference ligand arachidonic acid displayed the strongest predicted binding affinity (–9.1 kcal/mol), forming hydrogen bonds with Arg596 and His600, residues known to be involved in substrate positioning within the catalytic site.

Among the docked metabolites of *Coriolopsis gallica*, oleic acid exhibited a highly favorable binding energy (–9.0 kcal/mol), closely comparable to that of arachidonic acid. Its interaction profile included hydrogen bonds with His550, Asn554, and His372, together with extensive hydrophobic contacts involving Phe177, Ala410, Lys409, and Val175, consistent with the accommodation of long-chain fatty acids within the hydrophobic channel of 5-LOX.

Linoleic acid also showed a strong predicted affinity (–8.6 kcal/mol) and interacted mainly through hydrophobic contacts with residues such as Phe555, Phe610, Phe169, and Val175, reflecting its structural compatibility with the enzyme’s lipid-binding tunnel.

In addition, palmitic acid and stearic acid displayed favorable binding energies (–8.8 and − 8.4 kcal/mol, respectively), with interaction patterns dominated by hydrophobic contacts involving Phe177, Leu607, Ala410, Ile406, and Val175, suggesting that saturated fatty acids may also occupy the active site through nonpolar stabilization.

The iridoid glycoside aucubin exhibited a moderate yet notable affinity (–7.5 kcal/mol), forming multiple hydrogen bonds with Lys409, Ser171, Asp176, and Val175, indicative of a more polar interaction mode within the enzyme cavity. In contrast, 2,4-di-tert-butylphenol showed the lowest predicted affinity (–5.8 kcal/mol), stabilized by hydrogen bonds with Asn554 and His367 and hydrophobic interactions with Phe177 and Ala410, consistent with its smaller and predominantly hydrophobic structure.

Taken together, the docking results indicate that several fatty acids identified in *Coriolopsis gallica*, particularly linoleic and oleic acids, display favorable predicted affinities toward both COX-2 and 5-LOX, consistent with their structural compatibility with lipid-binding enzyme cavities. In parallel, aucubin exhibited a distinct interaction profile characterized by multiple hydrogen bonds with polar residues, while 2,4-di-tert-butylphenol and other hydrophobic metabolites showed moderate affinities primarily driven by nonpolar contacts.

Rather than providing direct mechanistic evidence, these in silico findings offer comparative and hypothesis-generating insights into how chemically diverse constituents of *C. gallica* may interact with key inflammatory enzymes. When considered alongside the in vitro antioxidant and anti-denaturation assays, the docking analysis supports a multicomponent contribution to the observed biological effects, potentially arising from complementary interaction modes and additive or synergistic effects within the crude extract.

It should be emphasized that molecular docking provides a static approximation of ligand–protein interactions and primarily serves as a hypothesis-generating tool. While the observed binding energies and interaction patterns suggest a potential affinity of selected compounds toward COX-2 and 5-LOX, they do not constitute direct evidence of functional inhibition. The absence of molecular dynamics simulations, RMSD/RMSF analyses, and binding free-energy calculations (e.g., MM-PBSA/GBSA) represents a limitation of the present study. Future investigations integrating these approaches will be necessary to further validate the stability and energetic relevance of the predicted complexes.

## Conclusion

The present study demonstrates the significant pharmacological potential of *Coriolopsis gallica* as a source of natural bioactive compounds. Chemical profiling revealed a diverse array of metabolites, including fatty acids, iridoid glycosides, and phenolic compounds, which collectively contribute to the observed antioxidant and moderate anti-inflammatory activities in vitro. Molecular docking studies further supported these findings by suggesting plausible binding modes and interaction patterns of linoleic acid, aucubin, and 2,4-di-tert-butylphenol effectively interact with key inflammatory enzymes COX-2 and 5-LOX. Linoleic acid exhibited the strongest binding affinity, while aucubin and 2,4-di-tert-butylphenol contributed through hydrogen bonding and hydrophobic interactions. These in silico results provide hypothesis-generating insights that complement the in vitro anti-inflammatory assays, rather than definitive mechanistic evidence. Overall, the combined chemical, biological, and computational analyses highlight the potential of *Coriolopsis gallica* as a promising natural source of antioxidant and anti-inflammatory compounds, supporting its relevance in natural product research and drug discovery. Future studies should focus on in vivo validation, isolation of individual bioactive molecules, and advanced computational approaches, including molecular dynamics simulations, to further elucidate the mechanisms underlying the observed biological effects.

## Data Availability

No datasets were generated or analysed during the current study.
